# Plumbagin enhances antimicrobial and anti-biofilm capacities of chlorhexidine against clinical *Klebsiella pneumoniae* while reducing resistance mutations

**DOI:** 10.1128/spectrum.00896-24

**Published:** 2024-08-20

**Authors:** Haifeng Liu, Huanchang Chen, Zhexiao Ma, Ying Zhang, Shihang Zhang, Deyi Zhao, Zhuocheng Yao, Tieli Zhou, Zhongyong Wang

**Affiliations:** 1 Department of Clinical Laboratory, The First Affiliated Hospital of Wenzhou Medical University, Key Laboratory of Clinical Laboratory Diagnosis and Translational Research of Zhejiang Province, Wenzhou, Zhejiang, China; 2 School of Laboratory Medicine and Life Science, Wenzhou Medical University, Wenzhou, Zhejiang, China; Duke University, Durham, North Carolina, USA

**Keywords:** chlorhexidine, plumbagin, synergistic, anti-biofilm, *Klebsiella pneumoniae*

## Abstract

With the widespread misuse of disinfectants, the clinical susceptibility of *Klebsiella pneumoniae* (*K. pneumoniae*) to chlorhexidine (CHX) has gradually diminished, posing significant challenges to clinical disinfection and infection control. *K. pneumoniae* employs overexpression of efflux pumps and the formation of thick biofilms to evade the lethal effects of CHX. Plumbagin (PLU) is a natural plant extract that enhances membrane permeability and reduces proton motive force. In this study, we elucidated the synergistic antimicrobial activity of PLU in combination with CHX, effectively reducing the MIC of CHX against *K. pneumoniae* to 1 µg/mL and below. Crucially, through crystal violet staining and confocal laser scanning microscopy live/dead staining, we discovered that PLU significantly enhances the anti-biofilm capability of CHX. Mechanistically, experiments involving membrane permeability, alkaline phosphatase leakage, reactive oxygen species, and RT-qPCR suggest that the combination of PLU and CHX improves the permeability of bacterial inner and outer membranes, promotes bacterial oxidative stress, and inhibits oqxA/B efflux pump expression. Furthermore, we conducted surface disinfection experiments on medical instruments to simulate clinical environments, demonstrating that the combination effectively reduces bacterial loads by more than 3 log_10_ CFU/mL. Additionally, results from resistance mutation frequency experiments indicate that combined treatment reduces the generation of resistant mutants within the bacterial population. In summary, PLU can serve as an adjuvant, enhancing the anti-biofilm capability of CHX and reducing the occurrence of resistance mutations, thereby extending the lifespan of CHX.

**IMPORTANCE:**

As disinfectants are extensively and excessively utilized worldwide, clinical pathogens are progressively acquiring resistance against these substances. However, high concentrations of disinfectants can lead to cross-resistance to antibiotics, and concurrent use of different disinfectants can promote bacterial resistance mutations and facilitate the horizontal transfer of resistance genes, which poses significant challenges for clinical treatment. Compared with the lengthy process of developing new disinfectants, enhancing the effectiveness of existing disinfectants with natural plant extracts is important and meaningful. CHX is particularly common and widely used compared with other disinfectants. Meanwhile, *Klebsiella pneumoniae*, as a clinically significant pathogen, exhibits high rates of resistance and pathogenicity. Previous studies and our data indicate a significant decrease in the sensitivity of clinical *K. pneumoniae* to CHX, highlighting the urgent need for novel strategies to address this issue. In light of this, our research is meaningful.

## INTRODUCTION

Disinfectants were once employed to control the spread of bacteria and viruses, and it was believed that they were less likely to induce resistance (1). However, with a significant increase in usage during the pandemic, bacteria can now form thick biofilms to evade the lethal effects of disinfectants. *Nature Reviews Microbiology* reported that the clinical isolates’ susceptibility to disinfectants has gradually declined (2). There is an urgent need for innovative research to address this serious issue. Chlorhexidine (CHX), as a cationic biguanide disinfectant, is one of the most commonly used disinfectants, effective against Gram-negative bacteria, Gram-positive bacteria, and various fungi (3). CHX targets phospholipid molecules on bacterial cell membranes, disrupting the cell membrane and causing leakage of cellular contents, thereby achieving bacterial eradication (4). It has been reported that the combined use of CHX with natural terpenoids, such as thymol and farnesol, is effective against wound infections caused by Gram-positive bacteria and holds promise as an additive in commercial mouthwash and care solutions (5). Although the combinations of these plant extracts with CHX hold significant commercial and medical value, they have not demonstrated synergistic effects against Gram-negative bacteria. This has prompted the research focus to advance toward addressing Gram-negative bacteria. Simultaneously, excessive use of CHX has led to a decreased susceptibility of Gram-negative bacteria to it (6, 7).

*Klebsiella pneumoniae* (*K. pneumoniae*), one of the most common Gram-negative bacteria causing infections, possesses various robust antibiotic resistance mechanisms (8). An article published in *The Lancet Microbe* analyzed a significant correlation between *K. pneumoniae* and severe pneumonia, a respiratory infection (9). They stand as major pathogens in hospitals, posing a challenging dilemma for clinical antibiotic treatment (10). Although disinfectants are frequently employed to eliminate them, unfortunately, a decline in disinfectant sensitivity and the emergence of disinfectant-resistance strains of *K. pneumoniae* have become apparent. This escalating trend poses a substantial threat to public health (11, 12).

Plumbagin (5-hydroxy-2-methyl-1,4-naphthoquinone, PLU) is a naturally occurring green plant extract, widely present in plants of Ancestrocladaceae, Plumbaginaceae, Droseraceae, and Dioncophyllaceae families (13). PLU holds tremendous potential in cancer treatment, antifungal activities, regulation of bacterial virulence, and reduction of biofilm formation (14–16). Importantly, PLU exhibits significant synergistic effects with the positively charged antibiotic colistin (17). PLU may exert its effects by inhibiting bacterial mitochondrial electron transport and altering membrane permeability (18, 19). We found that CHX carries a higher positive charge, similar to colistin (20). This led us to explore whether PLU could restore antimicrobial susceptibility to CHX. Importantly, there is currently no literature reporting the combined effects of PLU with disinfectants.

Compared with developing entirely new disinfectants, the synergistic use of existing substances with traditional disinfectants is more valuable. Simultaneously, evaluating the impact of combined drug applications on the emergence of disinfectant-resistant mutants is crucial (21). Our study aims to assess the potential of PLU to serve as an adjunct to CHX, expanding its application significance through medical device surface disinfection and determining resistance mutation frequencies. We believe that this systematic research can contribute to addressing the issue of the reduced susceptibility of clinical isolates to CHX.

## MATERIALS AND METHODS

### Repeatability statement

All experiments in this study were replicated three or more times.

### Materials and reagents

Most of the materials used in this study are listed in Table S1, and specific materials and instrument models are explained at their respective positions in the manuscript. Unless otherwise specified, the monotherapy concentrations in the monotherapy groups are the same as the monotherapy concentrations in the combination groups. For real-time fluorescence quantitative PCR (RT-qPCR), primer sequences can be found in Table S2. For polymerase chain reaction (PCR), the primer sequences are available in Table S3.

### Strains and growth

The strains used in this study were isolated from clinical patients in various departments of the First Affiliated Hospital of Wenzhou Medical University. Additional background information on the strains can be found in our previous study (22). Additionally, ATCC 700603 was procured from the National Collection of Clinical Strains (NCCL) in the United States as a standard strain. Matrix-assisted laser desorption/ionization time-of-flight mass spectrometry (MALDI-TOF/MS; BioMérieux, Lyon, France) was employed to identify all clinical strains. Isolated strains were cryopreserved in Luria-Bertani (LB) broth supplemented with 30% glycerol at −80°C for subsequent research. It is noteworthy that even after repeated subcultures, the strains maintained stable minimum inhibitory concentration (MIC) values.

### Antimicrobial susceptibility testing

We employed the microbroth dilution method to determine the MICs of CHX and PLU for all experimental strains, aiming to assess the strains’ susceptibility to the drugs (Table S4). The experimental procedure was conducted as previously described with some minor modifications (23). In brief, 96-well plates containing Mueller-Hinton broth supplemented with cation-adjusted Mueller-Hinton broth (CAMHB) were prepared, featuring a series of drug concentrations with geometric dilutions. Subsequently, 100 µL of bacterial suspension (1.5 × 10^6^ CFU/mL) was inoculated into each well of the plates, followed by incubation at 37°C for 16–18 hours. The minimum drug concentration at which there was no observable bacterial growth by the naked eye was defined as the MIC.

### Checkerboard assays

We employed the checkerboard assays (24) to determine whether the combination of CHX and PLU exhibits a synergistic effect. In essence, both drugs were diluted in CAMHB in a 96-well plate, resulting in a series of geometrically diluted drug concentrations. CHX was diluted along the horizontal axis, whereas PLU was gradually diluted along the vertical axis. Subsequently, 100 µL of bacterial suspension (1.5 × 10^6^ CFU/mL in PBS) was inoculated into each well. The 96-well plate was then incubated at 37°C for 16–18 hours, and the results were observed. To evaluate the synergy between CHX and PLU, the fractional inhibitory concentration index (FICI) was calculated. The formula for FICI is as follows: FICI = FIC_CHX_ + FIC_PLU_ = (MIC_CHX combined PLU_ / MIC_CHX alone_) + (MIC_PLU combined CHX_ / MIC_PLU alone_). The interpretation criteria for FICI values are defined as follows: FICI ≤0.5 indicates a synergistic effect (25).

### Time-kill assays

To elucidate the temporal dynamics of the synergistic antimicrobial efficacy of CHX and PLU combination, we conducted time-kill assays (26). Simply put, eight strains of bacteria (initial concentration of 1.5 × 10^6^ CFU/mL) were inoculated into 20 mL of CAMHB. Subsequently, different strains were treated with drug concentrations corresponding to their respective 2 × FICI values. All treatment groups were cultured at 37°C with agitation at 180 rpm. At various time points (0, 2, 4, 6, 12, and 24 hours), culture samples were taken and plated on LB agar for colony counting.

### Scanning electron microscopy

To observe bacterial morphology, silicon chips (3 × 3 mm) were placed in a 24-well plate to provide a flat surface for bacterial attachment (27). Subsequently, silicon chips were incubated in 24-well plates containing 1 mL of LB broth with a bacterial density of 1.5 × 10^6^ CFU/mL and drug concentration at 0.5 × FICI for 24 hours at 37°C. Afterward, silicon chips were rinsed three times with PBS, fixed in 2.5% glutaraldehyde at low temperatures for 15 minutes, and dehydrated in gradually increasing ethanol concentrations (30%, 50%, 70%, 80%, 90%, and 100%) for 10 minutes each. Following air-drying, the samples were gold-coated and observed using scanning electron microscopy (SEM) (Hitachi SU8010, Japan).

### Crystal violet stain

For the biofilm formation inhibition experiment, the procedures were as previously described (28). Simply put, a bacterial suspension with a concentration of 1.5 × 10^6^ CFU/mL was added to a 96-well plate in a volume of 100 µL, followed by the addition of drugs at concentrations corresponding to 0.5 × FICI for each strain. After co-incubation for 24 hours, the planktonic bacteria were discarded, and the wells were washed twice with PBS. Subsequently, the biofilm was air-dried and fixed. Staining and destaining were performed using 1% crystal violet solution and ethanol, respectively. Finally, the absorbance at 595 nm for each well was measured using a microplate reader (BioTek, Synergy).

For the mature biofilm eradication experiment, the procedures were similar to the biofilm formation inhibition experiment, with the only difference being the cultivation of mature biofilms before adding the drugs, as previously described (29). The bacterial suspension (1.5 × 10^6^ CFU/mL, 200 µL) was added to a 96-well plate, and mature biofilms were formed by direct incubation at 37°C for 24 hours. After discarding the liquid in the wells and washing twice with PBS, drug treatments corresponding to 2 × FICI concentrations for each strain were added for 24 hours. Subsequent air-drying, staining, and destaining steps were the same as those in the biofilm formation inhibition experiment.

### Confocal laser scanning microscopy live/dead staining

We followed the procedures outlined previously (30) with some modifications. To clearly demonstrate PLU’s significant enhancement of CHX’s anti-biofilm capability, we introduced dual staining with SYTO 9 (100 μg/mL) and Propidium iodide (PI, 50 µg/mL) fluorescent dyes to label bacteria within the biofilm. In summary, FK2175 (1.5 × 10^6^ CFU/mL) was inoculated into LB broth containing drugs at 0.5× FICI concentrations. The mixture was transferred to a confocal dish and incubated at 37°C for 24 hours to allow the formation of static biofilms on the dish. Subsequently, the biofilm was washed twice with sterile PBS to remove planktonic bacteria, followed by dual staining with SYTO 9 and PI according to the specified procedures. After two additional washes with sterile PBS to remove any excess dye, the biofilm was imaged using confocal laser scanning microscopy (CLSM) (Leica, Japan).

SYTO 9 emits green fluorescence, indicating live bacteria, whereas PI emits red fluorescence, indicating dead bacteria. To reflect the thickness of the biofilm, we performed three-dimensional imaging, where the height along the Z-axis was determined by scanning from the point of the first fluorescence appearance (representing the top layer) to the point of fluorescence disappearance (indicating the bottom layer). The ratio of red to green fluorescence served as an indicator of drug efficacy against bacteria within the biofilm. The green fluorescence density among groups was used to assess the thickness and density of the biofilm.

### Determination of outer membrane permeability

We evaluated outer membrane permeability using the 1-N-phenyl naphthylamine (NPN) fluorescent dye (31). Initially, bacterial cultures were prepared by overnight shaking incubation in LB broth, followed by three washes with PBS and resuspension in PBS to an OD_600_ of 0.3–0.4. Each strain was treated with drugs at concentrations corresponding to 0.5× FICI values for 2 hours. After centrifugation at 4,000 rpm for 5 minutes to remove the drug solution, cells were resuspended in an NPN solution (30 µg/mL) and stained for 30 minutes at 37°C. Subsequently, fluorescence intensity was measured on a microplate reader (BioTek, Synergy) at an excitation wavelength of 350 nm and emission wavelength of 420 nm.

### Determination of inner membrane permeability

We assessed inner membrane permeability using the PI fluorescent dye (17). The bacterial liquid concentration, drug concentration, and treatment time were consistent with the determination of outer membrane permeability. Subsequently, cells were resuspended in a PI solution (50 µg/mL) and stained for 30 minutes at 37°C. Fluorescence intensity was then measured on a microplate reader (BioTek, Synergy) at an excitation wavelength of 535 nm and emission wavelength of 615 nm.

### Extracellular alkaline phosphatase activity assay

This study assessed the disruption level of bacterial membrane integrity by detecting extracellular alkaline phosphatase (ALP) activity (32). Bacterial cultures with an OD_600_ of 0.5 were suspended in phosphate buffered saline (PBS), CHX, PLU, and CHX + PLU combinations for 6 hours, with drug concentrations corresponding to 0.5 × FICI values. Subsequently, reagents were added according to the kit instructions, and the OD_510_ absorbance was measured using a microplate reader. Changes in absorbance indicated ALP leakage due to membrane integrity disruption.

### Reactive oxygen species detection

The level of intracellular reactive oxygen species (ROS) in bacteria can be quantified using the corresponding assay kit, reflecting the oxidative stress level in response to drug stimulation (33). In brief, bacterial cultures with OD_600_ of 0.3–0.4 were prepared, loaded with a DCFH-DA probe for 30 minutes, and then incubated in the dark with different drugs for 2 hours. Immediately, the fluorescence intensity was measured using a microplate reader (BioTek, Synergy) with an excitation wavelength of 488 nm and an emission wavelength of 525 nm. The drug concentration was set at 0.5 × FICI values.

### Membrane potential detection

We employed the DiSC3(5) dye to detect changes in membrane potential, where increased fluorescence intensity indicates membrane depolarization (34). However, we made slight modifications to the experimental procedure. In essence, a 5 µM solution of DiSC3(5) was added to the bacterial suspension with OD_600_ = 0.5, loaded for 30 minutes, and then centrifuged at 4,000 rpm/5 min to remove excess dye. The cells were resuspended in drug solutions corresponding to 0.5 × FICI values and incubated for 30 minutes, followed by immediate measurement of fluorescence intensity using a multifunctional microplate reader (BioTek, Synergy) with an excitation wavelength of 622 nm and an emission wavelength of 670 nm.

### RT-qPCR and PCR

For the RT-qPCR detection of *oqxA/B* efflux pump gene expression, the method was conducted as previously described (35). In this experiment, *rpoB* was used as the housekeeping gene, and the 2^–ΔΔCt^ method was employed to calculate the *oqxA/B* gene expression levels. The protocol followed the manufacturer’s recommendations, and cDNA was reverse-transcribed and obtained using the Bacterial RNA Miniprep Kit and RevertAid First Strand cDNA Synthesis Kit. Subsequently, amplification was performed using the TB Green Premix Ex Taq II (Tli RNaseH Plus) kit. For RT-qPCR, primer sequences can be found in Table S3.

For PCR, the primer sequences are available in Table S4. The PCR program was set as follows: 34 cycles for *oqxA* with conditions of 94°C for 45 seconds, 57°C for 45 seconds, and 68°C for 60 seconds, and 32 cycles for *oqxB* with conditions of 94°C for 45 seconds, 64°C for 45 seconds, and 72°C for 60 seconds.

### Surface disinfection of medical devices

In this study, we utilized contaminated needles to simulate contaminated medical devices (36), assessing whether PLU could enhance the practical bactericidal effect of CHX against bacteria adhering to medical instruments. Simply put, a bacterial suspension with a concentration of 10^6^ CFU/mL was prepared as the initial contaminating bacterial load. Clean needles were immersed in the bacterial suspension for 1 hour, allowing bacteria to adhere to the needles. Subsequently, the needles were removed and air-dried for 1 hour before being subjected to a 2-hour treatment with drugs at concentrations corresponding to the FICI values. The needles were thoroughly washed with PBS, and the eluate was immediately plated onto blank LB agar plates for spread plating. After 16–18 hours of incubation, the plates were observed, and the bacterial colony counts were recorded.

### Determination of resistance mutation frequency

We combined the approaches of two existing literature with some modifications (36, 37). The experimental strains (initial concentration of 1.5 × 10^6^ CFU/mL) were cultured overnight in CAMHB and then diluted 1:1,000 into 1.5 mL CAMHB containing subinhibitory concentrations of drugs (e.g., combined concentration of 0.5 × FICI value). After 24 hours of growth, the culture was serially diluted by factors of 10. Subsequently, 100 µL of each dilution was plated on both blank LB agar plates and LB agar plates containing 64 µg/mL of CHX (the cutoff value for CHX). Following overnight incubation, colony counting was performed on each plate, and the average was calculated from three repetitions to determine the frequency of drug-resistant mutations. The calculation method was as follows: (number of colonies on the plate with CHX/number of colonies on the blank plate) × 100%. Statistical differences were assessed using one-way analysis of variance (ANOVA), and *P* values were calculated for comparison. This reflects the probability of a drug-resistant subpopulation within the total population in response to drug stimulation.

### Statistical analysis

The statistical analysis and graphical representation in this study were conducted using Prism 9.0 software (GraphPad Software, LLC; San Diego, California, USA). Data were presented as the mean ± standard deviation of at least three replicates, derived from at least three independent experiments. Statistical analyses employed Student’s *t*-test or ANOVA, with the significance level set at *P* < 0.05. The correlation between *P*-values and asterisks is as follows: *: *P* < 0.05, **: *P* < 0.01, ***: *P* < 0.001, ****: *P* < 0.0001.

## RESULTS

### Detection of antimicrobial susceptibility

Using the microbroth dilution method, we determined the susceptibility of 200 non-repeated clinical isolates of *K. pneumoniae*. The MIC distribution of CHX against these isolates ranged from 0.5 to 64 µg/mL, with MIC_50_ and MIC_90_ both at 16 µg/mL. Based on the MIC distribution, we selected the top 10 non-repeated clinical isolates with the highest MIC for CHX for further experimentation. These isolates represented a batch of bacteria clinically known to be more tolerant to CHX. Since there is no defined resistance breakpoint for CHX, we adopted the epidemiological cutoff values reported in the literature, using MIC ≥64 µg/mL as the standard for CHX resistance (6). Isolates with MIC values of 16 µg/mL and 32 µg/mL were defined as strains with reduced susceptibility to CHX. Following antimicrobial susceptibility testing, one strain among the 10 experimental strains showed resistance to CHX, whereas the remaining nine exhibited significantly reduced susceptibility to CHX (Table S1). Additionally, PLU, a non-antimicrobial compound derived from plants, demonstrated MIC values ≥ 256 µg/mL against all bacteria when applied individually, showing no apparent antimicrobial activity. Moreover, ATCC 700603 was used as a standard strain for experimental quality control.

### Synergistic effect determined by checkerboard assays

The Checkerboard assays revealed a significant synergistic effect of the combination of CHX and PLU on clinical isolates exhibiting reduced susceptibility to CHX. Importantly, PLU significantly lowered MICs of CHX by 16–64 times, markedly enhancing bacterial susceptibility to CHX (Table 1).

**TABLE 1:** Antimicrobial susceptibility of chlorhexidine and plumbagin single or in combination against clinical isolates used in this study^*a*^

Strains	Monotherapy MIC (μg/mL)		Combination MIC (μg/mL)		FICI	Interpretation
	CHX	PLU	CHX	PLU		
FK2007	16	≥256	1	16	≤0.125	Synergistic
FK2027	32	≥256	1	16	≤0.09375	Synergistic
FK2039	32	≥256	1	16	≤0.09375	Synergistic
FK2046	16	≥256	0.5	16	≤0.09375	Synergistic
FK2128	32	≥256	1	16	≤0.09375	Synergistic
FK2157	32	≥256	1	16	≤0.09375	Synergistic
FK2160	32	≥256	0.5	16	≤0.07813	Synergistic
FK2165	32	≥256	1	16	≤0.09375	Synergistic
FK2175	64	≥256	1	16	≤0.07813	Synergistic
FK2176	32	≥256	1	16	≤0.09375	Synergistic

^*a*^ FICI, fractional inhibitory concentration index.

Furthermore, we provide a standardized explanation for the concentrations used in different groups throughout subsequent experiments. In general, unless specified otherwise, the PBS group in each experiment serves as the blank or negative control. The concentrations in the CHX or PLU monotherapy groups align with those in the corresponding monotherapy concentrations of the combination group. Additionally, the COM group in each experiment is an abbreviation for combination, indicating the combination treatment group.

### Time-dependent killing analysis

**Fig 1:**
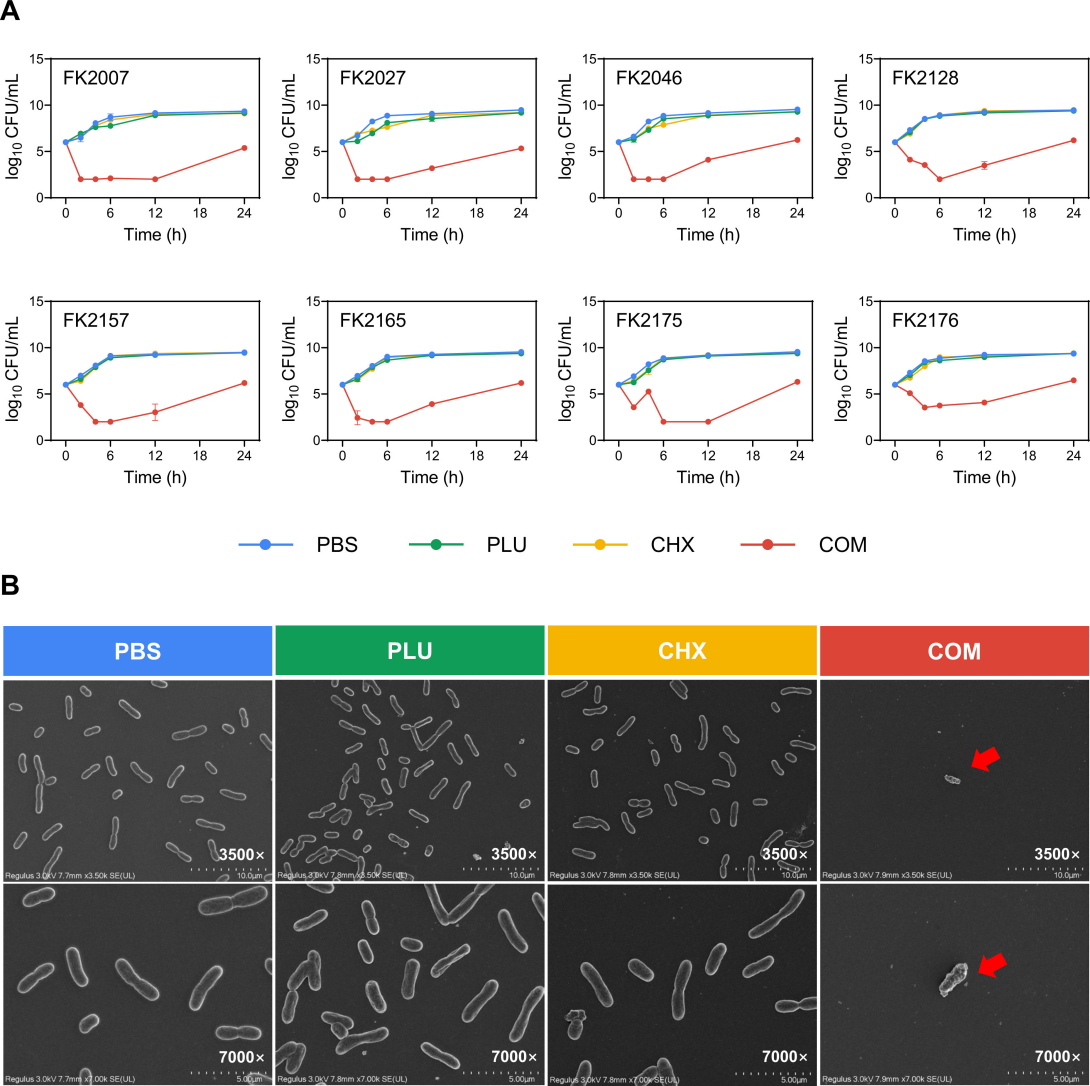
*In vitro* antimicrobial phenotypic experiments of the synergistic action of CHX and PLU. (**A**) Time-kill assays of bacteria treated with a combination or individually. (**B**) SEM images of bacteria treated with a combination or individually. Red arrows indicate abnormal bacterial morphology, with uneven surfaces, distortion, and damage to the cell membrane. COM refers to the combination group.

In order to investigate the time kinetics of the combined effect of CHX and PLU on bacterial eradication, we randomly selected eight representative strains for a time-kill assay. Due to the relatively close FICI values and the core concept of reducing the use of CHX, the concentrations in the experiment were uniformly set at CHX 1 µg/mL and PLU 32 µg/mL (approximately two times the FICI value). As shown in Fig. 1A, with PBS as a control, bacteria exhibited normal growth when CHX and PLU were applied individually, indicating that bacteria were not affected by their individual application. However, the combination of CHX and PLU showed bactericidal activity within 0–6 hours, rapidly reducing the bacterial count, and significantly decreasing the bacterial count by more than 3 log_10_ CFU/mL within 24 hours. These findings indicate that the combination of CHX and PLU can effectively eliminate strains with reduced susceptibility to CHX at low concentrations.

### SEM

As depicted in Fig. 1B, with PBS serving as the control, at magnifications of 3.5 K and 7.0 K, bacteria subjected to either CHX or PLU alone showed no significant differences in quantity or morphology compared with the PBS group. This indicates that bacteria are not significantly affected by the two individual drugs at low concentrations. In the combination group, due to the synergistic action of CHX and PLU, there was a noticeable reduction in bacterial quantity, and individual bacterial morphology exhibited apparent abnormalities, as indicated by the red arrows in the figure. Bacterial cell membranes appeared uneven, twisted, and damaged. This visual evidence reveals the effective destructive action of the CHX and PLU combination on strains with reduced susceptibility to CHX, supporting the results obtained from the Checkerboard assays and time-dependent killing analysis.

### Evaluation of anti-biofilm capability

**Fig 2:**
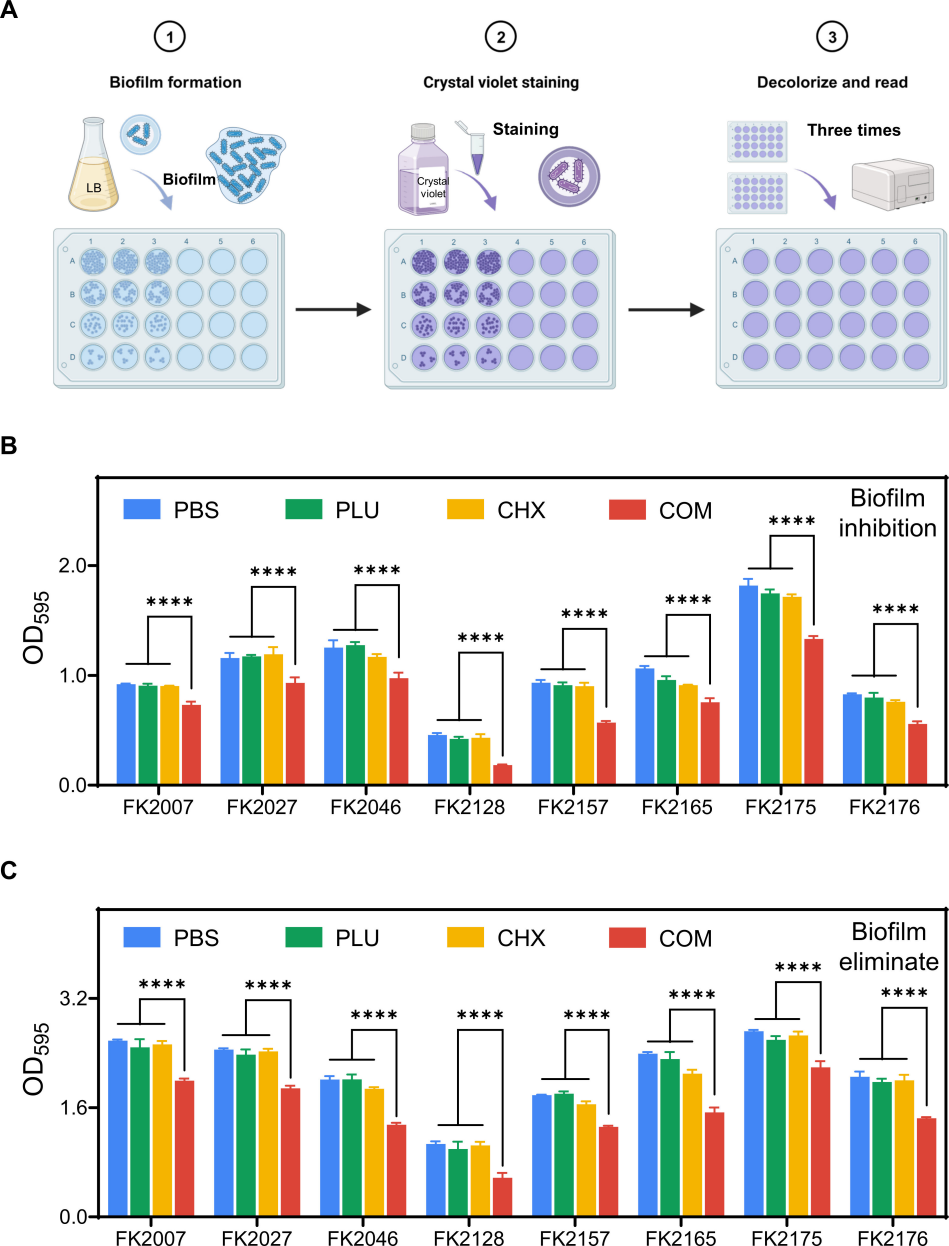
Crystal violet staining confirmed that PLU significantly enhances CHX’s anti-biofilm capability. (**A**) Simplified procedure of the crystal violet staining method. (**B**) Biofilm formation inhibition experiment. (**C**) Mature biofilm elimination experiment. COM refers to the combination group.

For the crystal violet staining method, as depicted in Fig. 2A, a simplified procedure for crystal violet experiments is shown. We selected the eight strains used in the time-dependent killing analysis for the experiments, conducting biofilm formation inhibition and mature biofilm eradication experiments. The statistical differences in Fig. 2 are shown as: ****, *P* < 0.0001. In the biofilm formation inhibition experiment, we uniformly chose sub-inhibitory concentrations at 0.5 × FICI for treatment. The results in Fig. 2B indicate that similar to the control group, there were no significant differences in the biofilm-forming ability of the strains treated with single drugs. However, the absorbance in the combination treatment group significantly decreased, suggesting that the combination treatment can effectively inhibit bacterial biofilm formation. In the mature biofilm eradication experiment, we uniformly chose drug concentrations at 2 × FICI. The results in Fig. 2C show that the combination treatment can significantly eliminate bacterial biofilm, a task that cannot be accomplished by the separate treatments of CHX or PLU alone. These results indicate that PLU imparts stronger anti-biofilm capability to CHX, which is dual-purpose, inhibiting early biofilm formation and eradicating mature biofilms.

### Visualization of anti-biofilm capability through CLSM live/dead staining

**Fig 3:**
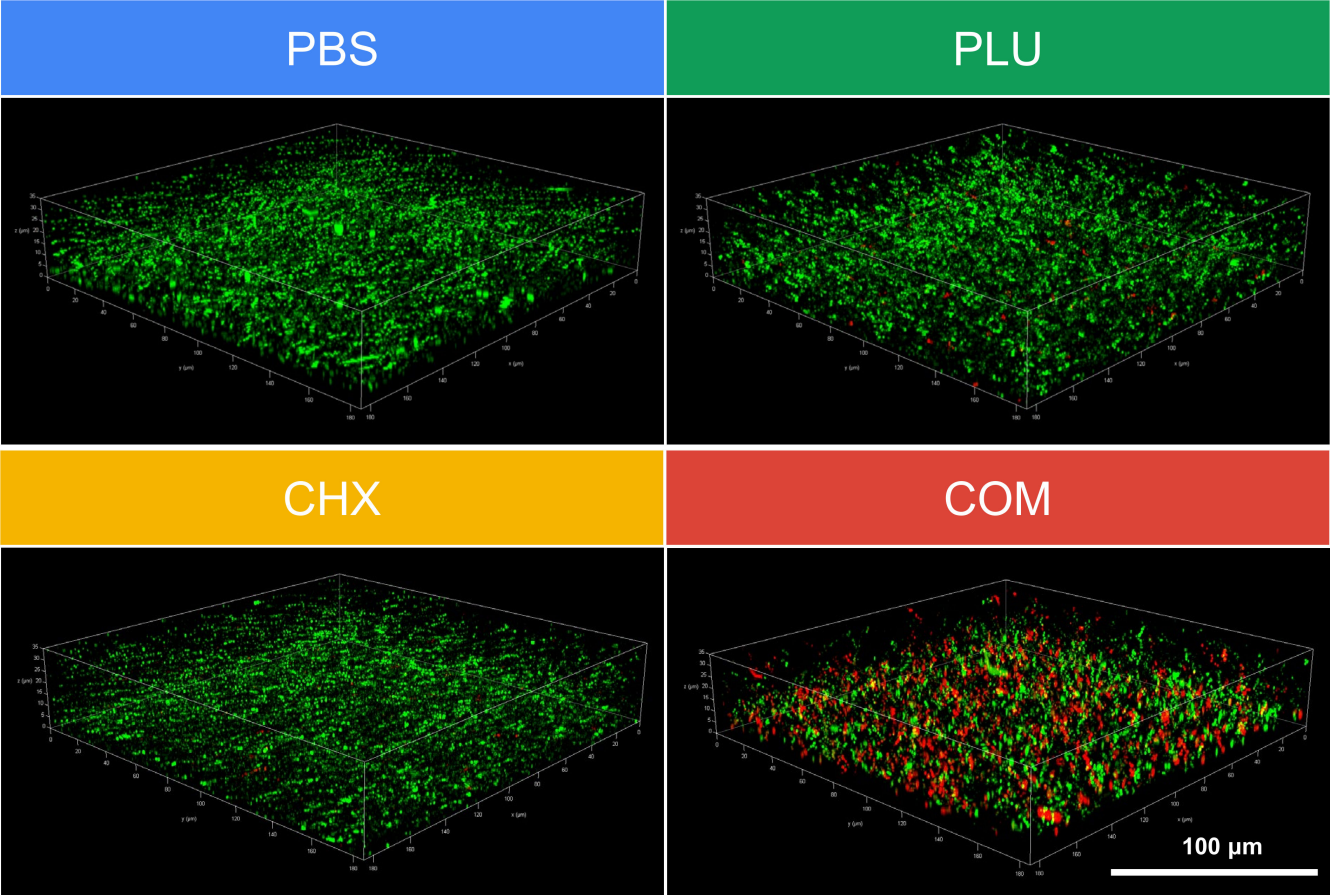
CLSM live/dead staining visualizes the enhancement of CHX anti-biofilm capability by PLU. COM refers to the combination group.

In the CLSM experiment, green fluorescence indicated live bacteria within the biofilm, whereas red fluorescence represented dead bacteria within the biofilm. As shown in Fig. 3, the combination treatment at sub-inhibitory concentrations of CHX and PLU led to a visibly looser density of green fluorescence compared with the other three groups, suggesting the inhibition of bacterial biofilm growth. Additionally, only the combination treatment group at sub-inhibitory concentrations exhibited a significant increase in red fluorescence, revealing the collective death of bacteria hidden within the biofilm under the pressure of combination treatment. Based on these findings, we posit that the superior anti-biofilm capability of the CHX and PLU combination, compared with monotherapy treatments, stems from the enhanced ability of the combination to facilitate the entry of CHX into bacteria, effectively eliminating bacteria within the biofilm to counteract biofilm formation. PLU endows CHX with the capability to dismantle the thick biofilm crucial for bacterial survival.

### Determination of outer membrane permeability

**Fig 4:**
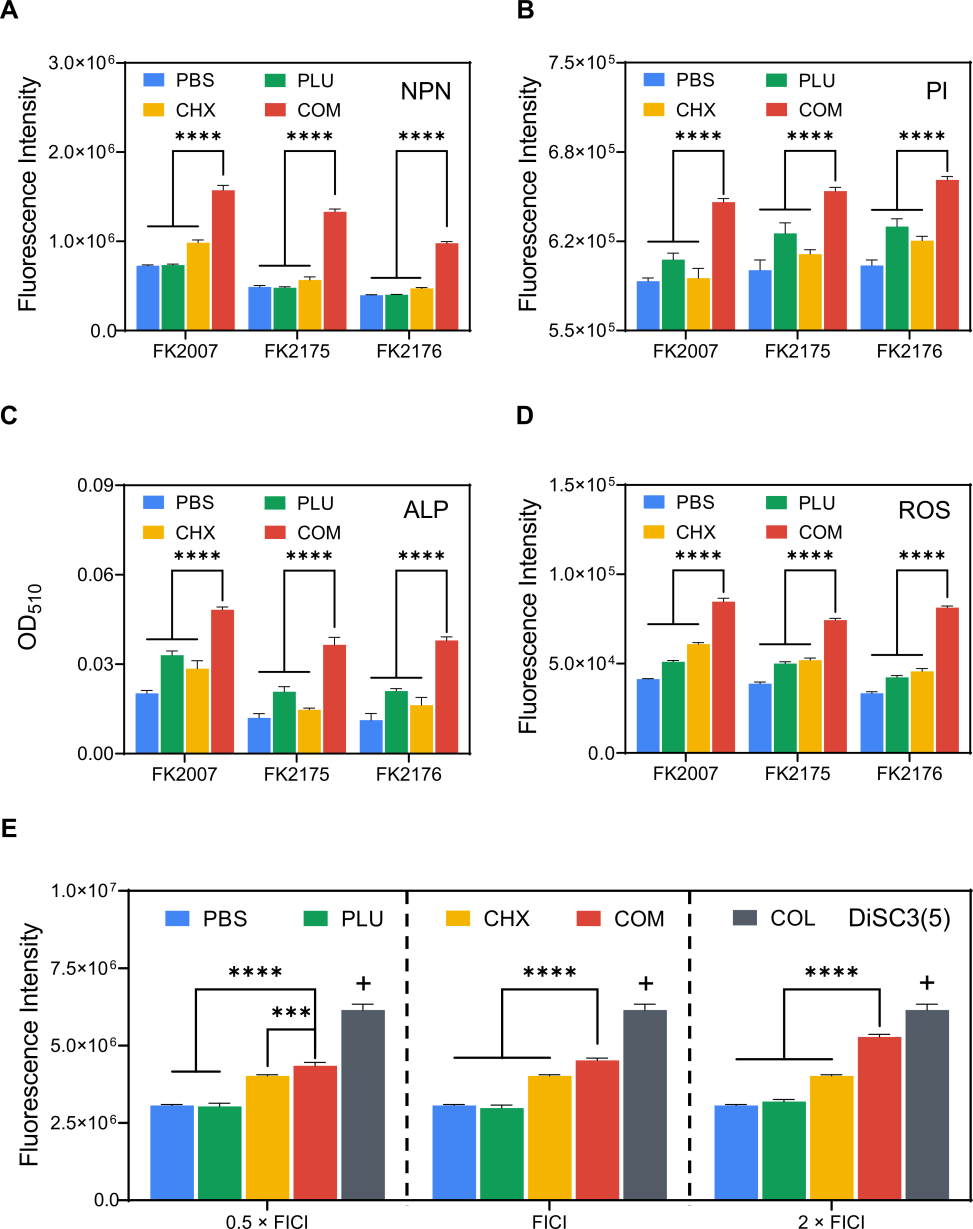
Possible mechanisms of the synergistic antimicrobial action of CHX and PLU. (**A**) NPN fluorescence probe detects bacterial outer membrane permeability. (**B**) PI fluorescence probe assesses bacterial inner membrane permeability. (**C**) Extracellular ALP activity assay evaluates bacterial membrane integrity. (**D**) Quantitative assay of ROS levels detects ROS production and bacterial oxidative stress. (**E**) DiSC3(5) fluorescence probe assesses changes in membrane potential and depolarization levels. Colistin (COL) serves as a positive control. COM refers to the combination group.

To explore the specific antimicrobial mechanisms of CHX and PLU in combination, we selected FK2007, FK2175, and FK2176 as experimental strains, all treated at sub-inhibitory concentrations (0.5 × FICI). We hypothesized that PLU could alter the permeability of bacterial inner and outer membranes, facilitating easier entry of CHX into bacteria and reducing the MIC. For the determination of the combined effect of CHX and PLU on outer membrane permeability, we employed the hydrophobic fluorescent dye NPN for fluorescence quantification. When changes or damage occurs in outer membrane permeability, NPN binds to the hydrophobic layer in the outer membrane, resulting in fluorescence. As illustrated in Fig. 4A, the fluorescence intensity of the combination treatment group was significantly higher compared with the control and monotherapy groups, indicating a substantial enhancement in bacterial outer membrane permeability. The statistical differences in the Fig. 4 are shown as: ***, *P* < 0.001, ****, *P* < 0.0001.

### Determination of inner membrane permeability

PI is a fluorescent dye used to detect inner membrane permeability or cell viability. Under normal conditions, PI cannot penetrate the bacterial inner membrane, binding to its internal DNA. However, when inner membrane permeability increases or bacteria die, PI can bind to DNA and emit fluorescence. As shown in Fig. 4B, the fluorescence intensity significantly increased in the combination treatment group of CHX and PLU compared with the other three groups. This enhancement is attributed to the increased membrane-penetrating ability conferred by PLU to CHX.

### ALP leakage assay

The extracellular ALP activity assay is commonly employed to assess bacterial membrane integrity. The experimental strains and drug concentrations were consistent with the outer membrane permeability assay. As shown in Fig. 4C, the absorbance in the CHX and ALP combination treatment group increased compared with the other three groups. We attribute this increase to enhanced extracellular ALP activity, indicating that the combination treatment can disrupt membrane integrity.

### ROS quantification

The DCFH-DA fluorescent dye was used to detect intracellular ROS accumulation, which is positively correlated with the degree of bacterial oxidative stress. As depicted in Fig. 4D, consistent with our expectations, the combination of CHX and PLU significantly increased intracellular ROS levels in bacteria, as evidenced by a marked enhancement in fluorescence intensity. Although both CHX and PLU monotherapy treatments also led to a certain degree of ROS elevation, the fluorescence intensity in the combination treatment group showed a distinctive and substantial increase compared with them, emphasizing the meaningful synergistic effect between the two agents. Therefore, we propose that the accumulation of ROS levels promotes bacterial oxidative stress, which may be one of the synergistic antimicrobial mechanisms of CHX and PLU.

### Membrane depolarization and proton motive force reduced

Many cationic drugs with high positive charges can induce changes in bacterial membrane potential, with colistin being a typical representative. CHX belongs to cationic surfactants, and its strong positive charge in solution can bind with the negatively charged membrane, causing depolarization of the cell membrane. To explore whether PLU can confer stronger membrane depolarization ability to CHX, we set up different concentration gradients of PLU (8, 16, and 32 µg/mL, corresponding to 0.5 × FICI, FICI, and 2 × FICI in the figure) with a fixed concentration of CHX (1 µg/mL) and used DiSC3(5) for membrane potential detection. As shown in Fig. 4E, we used PBS treatment as a negative control and 10 µg/mL colistin treatment as a positive control in the experiment. We found that even with the presence of PLU at 0.5 × FICI concentration, the combination group could still enhance fluorescence intensity by nearly double, showing a significant difference compared with the same concentration of CHX and PLU monotherapy. Moreover, this difference continued to increase with the elevation of PLU concentration, indicating that PLU can enhance the membrane depolarization ability of CHX, and this enhancement is positively correlated with concentration.

The efflux pump or extracellular cations can ultimately alter the bacterial electron transport chain (38). When membrane potential is depolarized, as electrons traverse the electron transport chain (e.g., NADH to NAD and FADH_2_ to FAD), they are transported outside the cell before binding with ADP to generate ATP. This process results in a decrease in proton concentration inside the membrane, leading to a weakening of the proton motive force. Concurrently, there is an accelerated absorption of positive charges by the cell. Therefore, based on the membrane potential results, we reasonably speculate that PLU may enhance the membrane depolarization ability of CHX because it induces a reduction in proton motive force inside the bacterial cell, thereby strengthening the adsorption of CHX to the bacterial membrane.

### RT-qPCR to detect the expression of potentially relevant efflux pump genes

**Fig 5:**
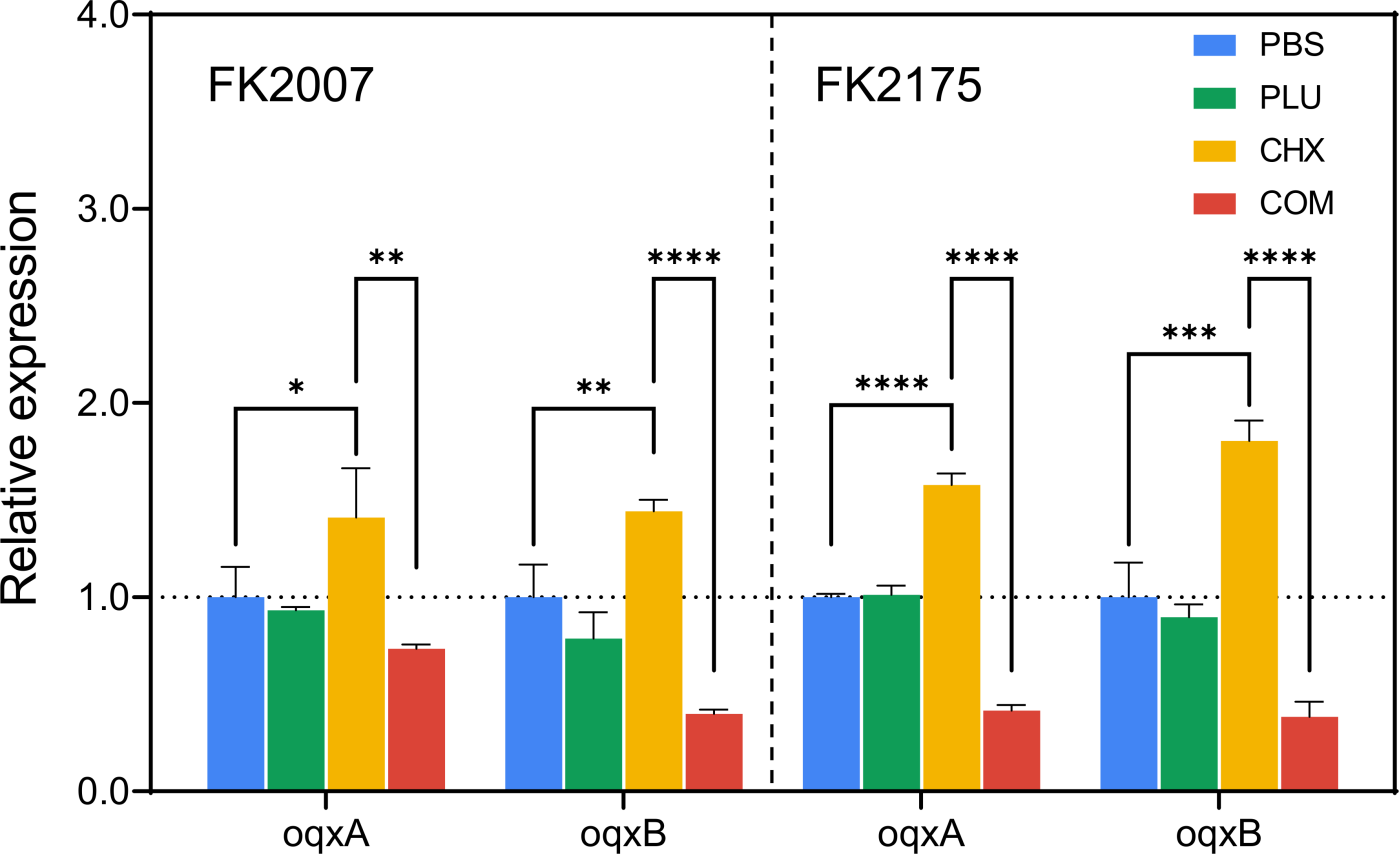
RT-qPCR was performed to measure the expression levels of the *oqxA/B* efflux pump genes in *K. pneumoniae*. COM refers to the combination group.

We conducted PCR experiments to verify the presence of the *oqxA/B* genes in the experimental strains, as shown in Fig. S1. The *oqxA* gene carriage rate was 100% (10/10), whereas the *oqxB* gene carriage rate was 30% (3/10). Subsequently, we selected two strains from the experimental bacteria that carried both *oqxA/B* genes for further RT-qPCR experiments, treating the bacteria with FICI concentrations of CHX and PLU. The statistical differences in Fig. 5 are shown as: *, *P* < 0.05, **, *P* < 0.01, ***, *P* < 0.001, ****, *P* < 0.0001. The results, as shown in Fig. 5, revealed that in FK2007 and FK2175, the expression levels of the *oqxA/B* genes increased under low CHX stimulation, showing a statistically significant difference compared with the PBS group. This difference was more pronounced in the CHX-resistant strain. Although the expression levels of the *oqxA/B* genes did not show a fault increase at low CHX levels, this may be attributed to the low concentration of experimental CHX (1 µg/mL) or the fact that CHX resistance mechanisms are orchestrated by the simultaneous expression of multiple efflux pumps. This is an avenue for future in-depth research and will not be further discussed here. Importantly, the combination of CHX and PLU significantly reduced the expression levels of the *oqxA/B* genes, suggesting that the combined treatment effectively inhibits the overexpression of the oqxA/B efflux pump. Based on these, we speculate that this exciting result may represent one of the mechanisms underlying the synergistic effects of the combined use of CHX and PLU on bacteria and biofilm.

**Fig 6:**
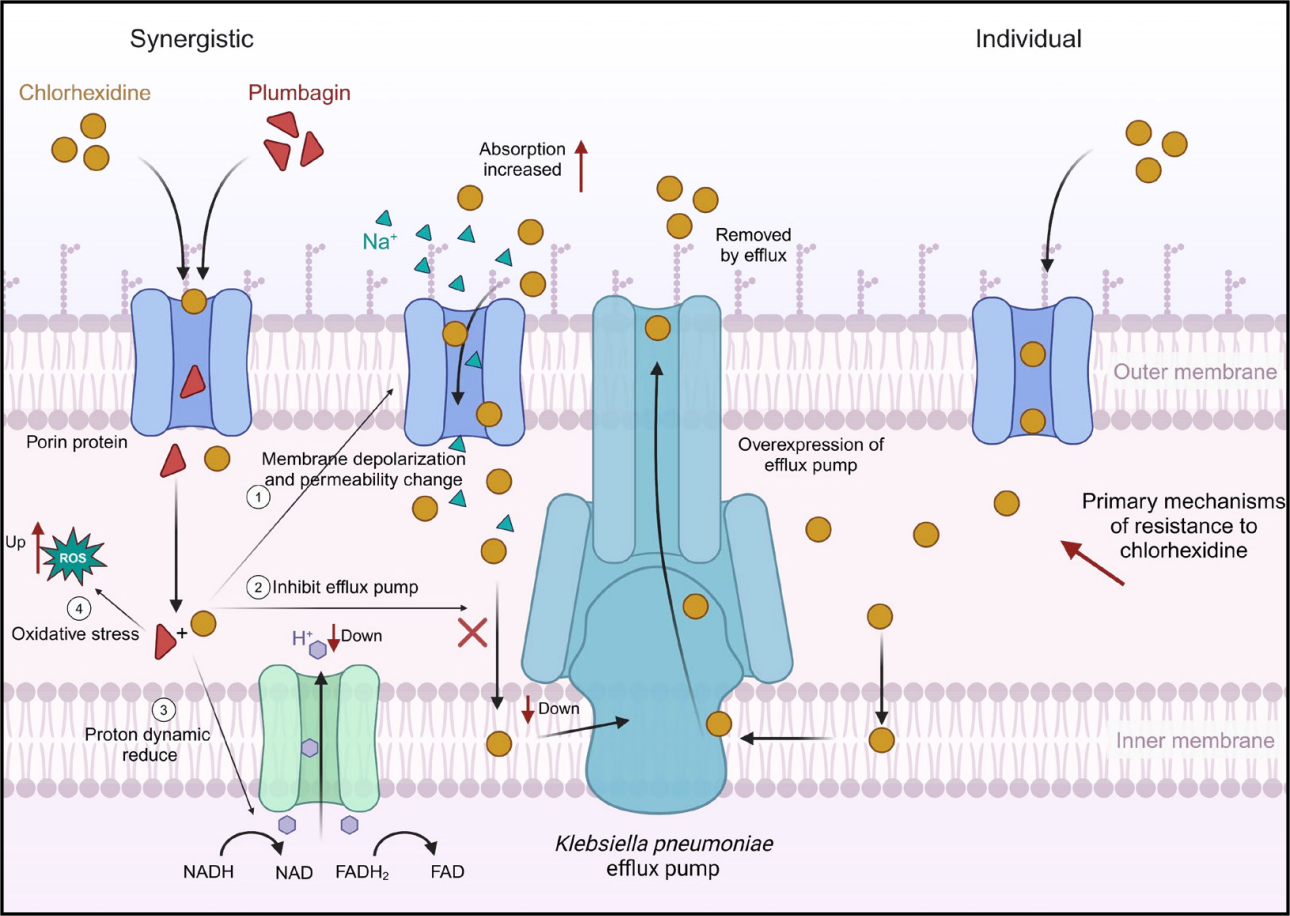
Diagram depicting the potential mechanisms and speculations on how PLU enhances the antimicrobial activity of CHX. The left half of the figure depicts the synergistic mechanism, whereas the right half illustrates the primary resistance mechanisms of CHX.

Finally, we concluded our speculations on the synergistic antimicrobial and antibiofilm mechanisms of CHX and PLU against *K. pneumoniae*. These conjectures, along with potential synergistic antimicrobial mechanisms, have been illustrated in Fig. 6.

### Simulates surface disinfection of medical devices

**Fig 7:**
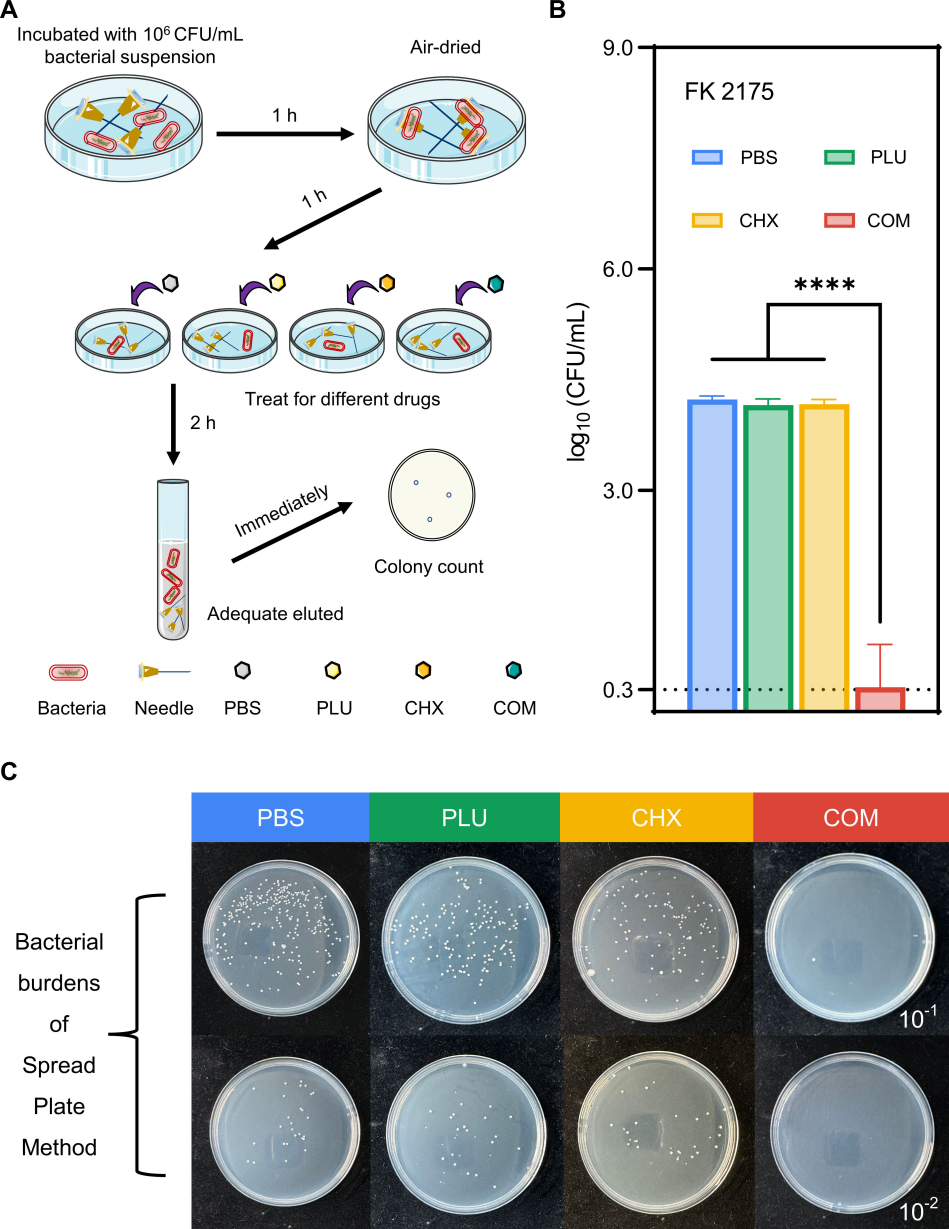
Medical device surface disinfection experiment simulating a disinfection environment. (**A**) Overview of the experimental procedure. (**B**) Bacterial colony counts. (**C**) Spread plate method visualizing the difference in colony loads with different drug treatments. COM refers to the combination group.

This simulation aimed to evaluate the sterilization capability of the CHX and PLU combination against medical instruments contaminated with CHX-resistant strains in clinical scenarios. The experimental procedure, outlined in Fig. 7A, involved contaminating disposable syringe needles with 10^6^ CFU/mL bacteria, allowing bacterial attachment to the needles. Subsequent drug treatments were applied, and after thorough washing with PBS, the eluate was dripped onto blank LB agar plates for overnight cultivation and colony counting. Results, depicted in Fig. 7B and C, indicated that a 2-hour combined treatment of CHX and PLU significantly reduced the bacterial count attached to disposable syringe needles by more than 3 log_10_ CFU/mL. The statistical differences in Fig. 7B are shown as: ****, *P* < 0.0001. Figure 7C visually presents the results of the spread plate method.

### Determination of resistance mutation frequency

The aim was to explore whether PLU can effectively reduce the overall resistance mutation frequency of sub-resistant bacteria after one treatment. Bacteria with resistance mutations were screened on LB agar plates containing 64 µg/mL (cutoff value) CHX. Likely due to the intrinsic sub-resistant state of the experimental strains, we observed the emergence of resistant strains after 24 hours of induction. The data in the table are the average values after three repeated experiments. The term 10^-n^ in the table represents the dilution factor. As shown in Table 2, after treatment with the combination of CHX and PLU, the resistance mutation frequencies for the three experimental strains were only 0.07%, 0.07%, and 0.29%, respectively. The slightly higher mutation rate of FK2176 compared with the other two strains may be attributed to its higher MIC. In contrast, with CHX treatment alone, the resistance mutation frequencies increased to 5.03%, 5.30%, and 6.23%, respectively. Through one-way ANOVA, these results showed significant statistical differences, with *P* value < 0.0001.

**TABLE 2:** The frequency of chlorhexidine-resistant subpopulation was observed following treatment with chlorhexidine alone or in combination with plumbagin^*a*^

Strains	Treatment	Colony count ( × dilution factor)		Mutation frequency	*P* value
		Blank plate	CHX plate		
FK2007	CHX	151.67 × 10^−2^	76.33 × 10^−3^	5.03%	<0.0001
	COM	116.33 × 10^−2^	8.00 × 10^−4^	0.07%	
FK2046	CHX	103.33 × 10^−2^	54.67 × 10^−3^	5.30%	<0.0001
	COM	101.67 × 10^−2^	7.33 × 10^−4^	0.07%	
FK2176	CHX	172.33 × 10^−2^	107.33 × 10^−3^	6.23%	<0.0001
	COM	168.00 × 10^−2^	48.67 × 10^−4^	0.29%	

^*a*^ Blank plate, LB agar plate; CHX plate, LB agar plate containing 64 µg/mL (cutoff value) chlorhexidine.

## DISCUSSION

Abundant research indicates that Gram-negative pathogens can persist in healthcare facilities for extended periods, leading to infections (39–41). This phenomenon transforms healthcare institutions into long-term bacterial reservoirs, providing opportunities for the emergence of drug-resistant strains (42). *K. pneumoniae*, as one of the most common infectious pathogens in clinical settings, has shown a continuous upward trend in recent infection rates (43). They often exhibit acquired resistance to multiple antibiotics, resulting in severe clinical infections (44). Some strains are highly virulent and capable of forming robust biofilms (45), posing significant challenges to clinical treatment and public health (46).

CHX, a cationic surfactant belonging to the biguanide class, is a widely used disinfectant in oral care, surgical tubing, and medical instruments (47). Despite the fact that the concentration of CHX in commercial disinfectant products is much higher than that used in this study, residual CHX still poses serious health risks (48, 49). In clinical practice, CHX is frequently employed to control the proliferation of *K. pneumoniae* (50). Unfortunately, with the widespread use of chlorine-containing disinfectants, the susceptibility of *K. pneumoniae* to CHX has gradually diminished, leading to the emergence of CHX-resistant strains (51, 52). Once individual bacteria breach the disinfectant barrier, the rapid horizontal spread of resistance genes within the bacterial population results in a swift decline in sensitivity, making disinfectants less reliable (53). Despite the option to increase the concentration of disinfectants continually, a substantial body of literature reports that high concentrations may prompt bacteria to develop antibiotic resistance, contributing to various forms of cross-resistance, and indirectly accelerating the road to antibiotic ineffectiveness (54, 55).

The phenomenon of CHX resistance may be associated with biofilm formation and overexpression of efflux pumps (11, 56). Sub-inhibitory concentrations of CHX can promote bacterial biofilm formation, subsequently leading to a decrease in antimicrobial susceptibility to CHX (57, 58). Surfaces of medical instruments such as surgical tubing and injection needles, as well as public health environments, often harbor biofilms that are challenging to clean. Bacteria within these biofilms can effectively evade the pursuit of residual CHX, thereby developing resistance (59, 60). Without exaggeration, the thick biofilm serves as a significant weapon for bacteria against CHX. Addressing this phenomenon, we believe that proposing strategies to enhance CHX’s anti-biofilm capabilities is urgently needed. When the current use of CHX can effectively eliminate bacteria within biofilms, the lifespan of CHX will be significantly extended. Referring to effective strategies against antibiotic resistance in clinical practice, the rapid and cost-effective option of combining different drugs is highlighted (61). Given the widespread use of CHX as a disinfectant, encompassing public environmental disinfection and personal hygiene products, we believe that low side effect, environmentally friendly natural plant extracts are suitable adjuvants. The combined use of natural plant extracts with disinfectants is meaningful, and research in this area can contribute to the development of novel health disinfectants or prolong the lifespan of existing disinfectants, contributing to public environmental health and personal hygiene.

Building upon the aforementioned information, we present the inaugural report on the synergistic antimicrobial and anti-biofilm effects of CHX and PLU, along with their mechanisms. We observed promising outcomes in both medical device surface disinfection and the reduction of resistance mutation frequency. The synergistic interaction between CHX and PLU is supported by checkerboard assays, time-kill assays, and SEM. Our previous research has confirmed that PLU exhibits no significant hemolytic toxicity to erythrocyte at concentrations ≥ 128 µg/mL and shows no noticeable toxicity to animal models at 64 and 32 µg/mL (17). Furthermore, literature indicates that PLU is non-toxic to A549 cells and HEK-293 cells at concentrations above 16 µg/mL and only exhibits mild toxicity at concentrations ≥ 32 µg/mL (62). Therefore, we believe the concentration of PLU (16 µg/mL) used in our study is safe. Additionally, crystal violet staining and CLSM demonstrate that PLU enhances the anti-biofilm effect of CHX. The application significance of this combination is further expanded through medical device surface disinfection and resistance mutation frequency determination. Although additional data may be necessary to substantiate the evidence supporting PLU’s ability to reduce the resistance mutation frequency in CHX-sensitive bacteria, we believe this aspect can pique the interest of researchers in relevant fields.

One of the resistance mechanisms of CHX involves the overexpression of efflux pumps. The oqxA/B efflux pump was initially associated with resistance to quinolone drugs, but literature also reports its involvement in low-level disinfectant resistance in *Escherichia coli* (63). The *oqxA/B* genes are typically present in multidrug-resistant bacteria, contributing to their enhanced resistance capabilities (64). Simultaneously, a previous article has reported that the detection rate of *oqxA* in carbapenem-resistant *K. pneumoniae* was as high as 60.9%, with *oqxB* detected in 17.2% of strains, and these strains exhibit stronger and broader resistance to disinfectants (65). However, there is no literature reporting the role of the oqxA/B efflux pump in low-level CHX resistance in *K. pneumoniae*. Meanwhile, the expression of the oqxA efflux pump in *K. pneumoniae* has been found to be positively correlated with biofilm-forming ability (66). Therefore, based on the positive results of the biofilm experiments and the resistance mechanism of CHX, we speculate that the oqxA/B efflux pump of *K. pneumoniae* may potentially contribute to or exacerbate resistance to low-level CHX. Although the resistance mechanisms involving efflux pumps are intricate, our discovery could potentially serve as a new target. Furthermore, we demonstrate that the combination of CHX and PLU significantly reduces its expression under low CHX pressure.

At the mechanistic level, we propose that altering membrane permeability, inducing membrane depolarization, and accumulating ROS contribute to the synergistic antimicrobial effects. We speculate that the downregulation of the *oqxA/B* efflux pump genes may be associated with the observed synergistic effects. Changes induced by PLU in membrane permeability and membrane potential are likely to enhance bacterial absorption of CHX, a hypothesis that can be further validated through more in-depth experiments in the future. Importantly, PLU may bolster CHX’s anti-biofilm capability by jointly suppressing bacterial growth within the biofilm through the inhibition of oqxA/B efflux pump expression. This is because efflux pumps and biofilms can mutually influence each other (66). Additionally, effective eradication of bacteria within the biofilm can be directly observed, particularly using CLSM.

These results indicate a significant advancement in the field of enhancing CHX’s antimicrobial effectiveness with medicinal plant extracts. This progress makes CHX more effective against *K. pneumoniae*, addressing the challenge of reduced susceptibility. This comprehensive study lays the foundation for future research in commercial applications and the development of disinfection methods for environmental and medical purposes.

## SUPPLEMENTAL MATERIAL

Supplemental material spectrum.00896-24-s0001.docx Fig. S1; Tables S1 to S4.

ASM does not own the copyrights to Supplemental Material that may be linked to, or accessed through, an article. The authors have granted ASM a non-exclusive, world-wide license to publish the Supplemental Material files. Please contact the corresponding author directly for reuse.
