# Supplementary material for: Plumbagin enhances antimicrobial and anti-biofilm capacities of chlorhexidine against clinical Klebsiella pneumoniae while reducing resistance mutations
Source: Microbiol Spectr. 2024 Aug 20;12(10):e00896-24. doi: 10.1128/spectrum.00896-24 (PMC11448042; doi:10.1128/spectrum.00896-24)
Supplement: Supplemental material — Fig. S1; Tables S1 to S4. [file spectrum.00896-24-s0001.docx]

**Supporting Information for**

**Plumbagin enhances antimicrobial and anti-biofilm capacities of chlorhexidine against clinical** ***Klebsiella pneumoniae* while reducing resistance mutations**

Haifeng Liu^a^, Huanchang Chen^a^, Zhexiao Ma^b^, Ying Zhang^a^, Shihang Zhang^a^, Deyi Zhao^b^, Zhuocheng Yao^a^, Tieli Zhou^a*^, Zhongyong Wang^a*^

^a^ Department of Clinical Laboratory, The First Affiliated Hospital of Wenzhou Medical University; Key Laboratory of Clinical Laboratory Diagnosis and Translational Research of Zhejiang Province, Wenzhou, Zhejiang, China

^b^ School of Laboratory Medicine and Life Science, Wenzhou Medical University, Wenzhou, Zhejiang, China

***** **Corresponding author:**

Zhongyong Wang (Corresponding author). Address: Department of Clinical Laboratory, The First Affiliated Hospital of Wenzhou Medical University; Key Laboratory of Clinical Laboratory Diagnosis and Translational Research of Zhejiang Province, Wenzhou, Zhejiang, China; Tel: +86-0577-8806-9595; Fax: +86-0577-8806-9595; E-mail: wangforever2000@163.com.

Tieli Zhou (Co corresponding author). Address: Department of Clinical Laboratory, The First Affiliated Hospital of Wenzhou Medical University; Key Laboratory of Clinical Laboratory Diagnosis and Translational Research of Zhejiang Province, Wenzhou, Zhejiang, China; Tel: +86-0577-8668-9885; Fax: +86-0577-8668-9885; E-mail: wyztli@163.com.

**Supplementary Information includes:**

- Fig. S1
- Table S1 – S4


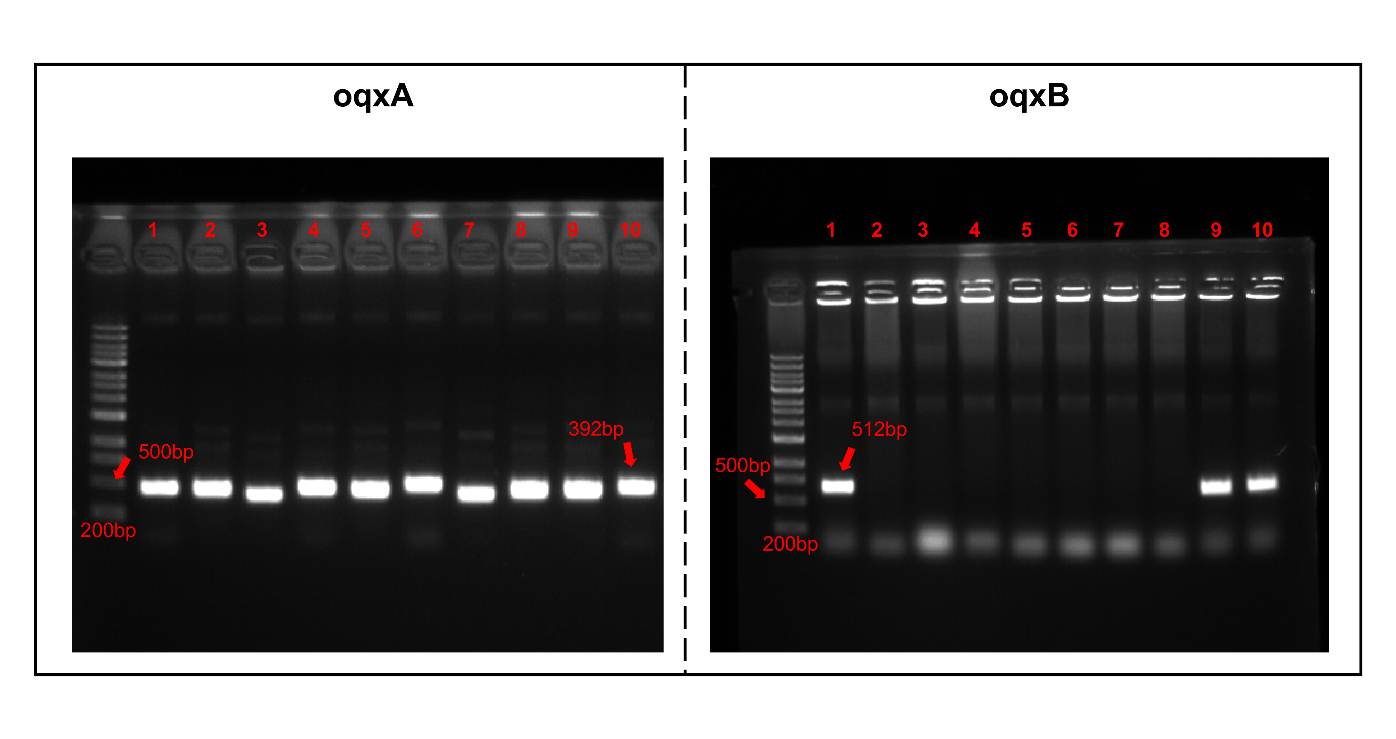
**Fig. S1** PCR results for the experimental strains carrying the *oqxA/B* efflux pump genes. The leftmost lane in both the oqxA and oqxB images corresponds to the marker. Lanes 1-10 correspond to the strains: FK2007, FK2027, FK2039, FK2046, FK2128, FK2157, FK2160, FK2165, FK2175, FK2176.

**Table S1** Main materials used in this study and the corresponding manufacturers.

| **Materials** | **Manufacturer** |
| --- | --- |
| Chlorhexidine acetate | MedChemExpress (China) |
| Plumbagin | Aladdin (Shanghai, China) |
| Glycerol | Thermo Fisher Scientific (America) |
| PBS 1× | Solarbio (Beijing, China) |
| Crystal violet | Solarbio (Beijing, China) |
| DiSC3(5) | MedChemExpress (China) |
| CCK-8 | Solarbio (Beijing, China) |
| Propidium iodide | Solarbio (Beijing, China) |
| SYTO 9 | Thermo Fisher Scientific (America) |
| 1-N-phenyl naphthylamine | Aladdin (Shanghai, China) |
| ROS assay kit | Beyotime (Shanghai, China) |
| ALP reagent kit | Solarbio (Beijing, China) |
| Bacterial RNA Miniprep Kit | Biomiga (Shanghai, China) |
| RevertAid First Strand cDNA Synthesis Kit | Thermo Fisher Scientific (America) |
| TB Green Premix Ex Taq II (Tli RNaseH Plus) (2×) | Takara (Japan) |
| Tli RNaseH Plus | Takara (Japan) |
| 2.5% glutaraldehyde | Servicebio (Hubei, China) |

**Table S2** Primers used to amplify mRNAs via RT-qPCR.

| **Typing** | **Gene** | **Forward primer**  **( 5′ - 3′ )** | **Reverse primer**  **( 5′ - 3′ )** | **Length (nt)** | **Annealing temperature (℃)** |
| --- | --- | --- | --- | --- | --- |
| RND family | *oqxA* | CGCAGCTTAACCTCGACTTCA | ACACCGTCTTCTGCGAGACC | 168 | 57 |
| RND family | *oqxB* | CGAAGAAAGACCTCCCTACC | CGCCGCCAATGAGATACA | 178 | 58 |
| Housekeeping | *rpoB* | AAGGCGAATCCAGCTTGTTCAGC | TGACGTTGCATGTTCGCACCCATCA | / | / |

**Table S3** Primers used to amplify DNAs via PCR.

| **Typing** | **Gene** | **Forward primer**  **( 5′ - 3′ )** | **Reverse primer**  **( 5′ - 3′ )** | **Size (bp)** |
| --- | --- | --- | --- | --- |
| RND family | *oqxA* | CTCGGCGCGATGATGCT | CCACTCTTCACGGGAGACGA | 392 |
| RND family | *oqxB* | TTCTCCCCCGGCGGGAAGTAC | CTCGGCCATTTTGGCGCGTA | 512 |

**Table S4** Background information regarding the isolates used in the experiment.

| **Strains** | **Separation** | **Sample** | **CHX MIC (μg/mL)** | **PLU MIC (μg/mL)** | **Resistance gene** |
| --- | --- | --- | --- | --- | --- |
| FK2007 | Neurosurgery | Sputum | 16 | ≥256 | *oqxA/oqxB* |
| FK2027 | Neurosurgery | Sputum | 32 | ≥256 | *oqxA* |
| FK2039 | Neurosurgery | Urine | 32 | ≥256 | *oqxA* |
| FK2046 | Thoracic surgery | Sputum | 16 | ≥256 | *oqxA* |
| FK2128 | Transplantation department | Urine | 32 | ≥256 | *oqxA* |
| FK2157 | Thoracic surgery | Sputum | 32 | ≥256 | *oqxA* |
| FK2160 | Thoracic surgery | Sputum | 32 | ≥256 | *oqxA* |
| FK2165 | Intensive care unit | Blood | 32 | ≥256 | *oqxA* |
| FK2175 | Neurosurgery | Urine | 64 | ≥256 | *oqxA/oqxB* |
| FK2176 | Cardiovascular medicine | Sputum | 32 | ≥256 | *oqxA/oqxB* |
| ATCC 700603 | / | / | 32 | ≥256 | *oqxA/oqxB* |
